# Novel weapon-aided plant protection in the underground battlefield

**DOI:** 10.1080/15592324.2024.2404808

**Published:** 2024-09-16

**Authors:** Seonghan Jang, Jin-Soo Son, Eric A Schmelz, Choong-Min Ryu

**Affiliations:** a Molecular Phytobacteriology Laboratory, Infectious Disease Research Center, KRIBB, Daejeon, South Korea; b Section of Cell and Developmental Biology, University of California, San Diego, La Jolla, CA, USA; c Department of Biosystems and Bioengineering, KRIBB School, University of Science and Technology, Daejeon, South Korea

**Keywords:** *Ralstonia solanacearum*, bacterial wilt, disease control, phytopathogen, plant protection

## Abstract

*Ralstonia solanacearum* and *R. pseudosolanacearum*, the causative agents of bacterial wilt, ranks as the second most devastating phytopathogens, affecting over 310 plant species and causing substantial economic losses worldwide. *R. solanacearum* and *R. pseudosolanacearum* infect plants through the underground root system, where it interacts with both the host and the surrounding microbiota and multiply in the xylem where bacteria cell and its polysaccharide product block the water transportation from root to aboveground. Currently, effective control methods are limited, as resistance genes are unavailable and antibiotics prove ineffective. In current Commentary, we review recent advancements in combating bacterial wilt, categorizing the approaches (weapons) into three distinct strategies. The physical and chemical weapons focus on leveraging sound waves to trigger crop immunity and reducing bacterial virulence signaling, respectively. The biological weapon employs predatory protists to directly consume *Ralstonia* cells in the root zone, while also reshaping the protective rhizosphere microbiome to fortify the plant. We believe that these novel methods hold the potential to revolutionize crop protection from bacterial wilt and inspire new era in sustainable agriculture.

*Ralstonia solanacearum* and *R. pseudosolanacearum*, soil-borne bacteria, are globally recognized as the second most notorious phytopathogen, responsible for bacterial wilt disease in over 310 plant species across 42 different families, causing severe economic losses ranging from 20–100%, depending on the crop species.^1^ Various traditional control strategies, including breeding, and physical and chemical treatments, have been employed to combat this devastating pathogen. Despite these efforts, effective management of bacterial wilt remains elusive.^1^ For instance, breeding for resistant plant varieties, while a feasible approach, is slow and labor-intensive, with resistance often breaking down after repeated cultivation in the field. Physical methods, such as disinfection, hot water treatment, and soil solarization, are effective but time-consuming and difficult to implement over large areas. Chemical treatments have not only led to the emergence of resistant *Ralstonia* strains but have also disrupted the native soil microbiome. Therefore, more efficient and environmentally friendly strategies capable of delivering the desired level of control are essential for eradicating this devastating disease. Recent advancements have opened up promising new avenues for bacterial wilt management. In this commentary, we categorize these innovative strategies into three main approaches: biological, chemical, and physical control weapons (Figure 1).

**Figure 1.**
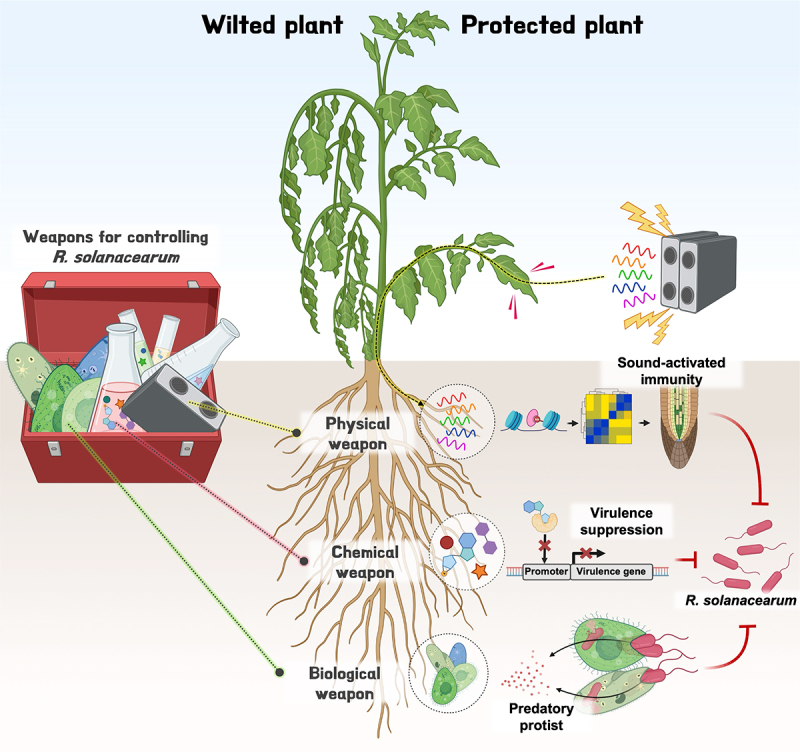
Novel strategies for bacterial wilt management.

Three new strategies for managing *R. solanacearum* are highlighted: physical, biological, and chemical approaches – each serving as a distinct “weapon” against the pathogen. The physical weapon utilizes 10 kHz acoustic sound vibrations to obstruct pathogen invasion by inducing epigenetic modifications in plant roots, which in turn trigger transcriptomic changes that enhance root immunity. The biological weapon employs ciliates from the genus *Colpoda*, predatory protists that mitigate bacterial wilt by preying on *R. solanacearum* cells. The chemical weapon includes the bacterial second messenger 2’,3’-cGMP and a cocktail of prebiotics, both of which suppress bacterial wilt symptoms by either downregulating bacterial virulence genes or inhibiting bacterial proliferation. These innovative tools show promise for practical field application, though further verification is required.

## Physical weapon: acoustic wave as an immune activator

Sound vibration (SV) has recently emerged as a promising physical weapon for controlling bacterial wilt disease.^2^ When *Arabidopsis* plants were exposed to SV (90 dB, 10 kHz), they exhibited a significant reduction in bacterial wilt symptoms. To explore whether SV combats bacterial wilt through epigenetic modifications in plants, Jung and colleagues performed an integrative analysis combining chromatin immunoprecipitation with microRNA sequencing. Their findings revealed that SV treatment led to elevated levels of the H3K27me3 modification in the promoter regions of genes associated with aliphatic glucosinolate biosynthesis and cytokinin signaling, leading to transcriptional changes that bolster plant immunity. In addition, the downregulation of miR397b under SV treatment upregulated its target lignin biosynthesis genes (*LAC2, LAC17*, and *IRX12*), which increased lignin content in the roots, thereby strengthening the plant cell walls and enhancing resistance to *R. solanacearum*. Further transcriptomic analysis of *Arabidopsis* plants exposed to 100-dB sound at a frequency of 1,000 Hz revealed increased expression of genes involved in various defense responses and salicylate-dependent signaling pathways. While research on using SV to control *R. solanacearum* is still in its early stages, further studies to optimize SV conditions for enhancing plant immunity could position SV as a sustainable alternative to chemical-based plant protection strategies.

## Biological weapons: predator protists and beneficial microbe assembly

Rhizosphere protists are widely recognized as crucial determinants of plant health.^3^ The first example involves the introduction of predatory protists on *R. solanacearum*-infected tomato plants cultivated under three fertilization regimes: conventional, organic, and bioorganic, each exhibiting different levels of bacterial wilt disease incidence.^4^ In this study, metabarcoding analyses revealed a significant negative correlation between predatory protists, especially the ciliophoran *Colpoda* OTU67, and bacterial wilt disease incidence (*p* = 0.007). *Colpoda* OTU67 also demonstrated the greatest explanatory power for the ratio of pathogen-suppressive to pathogen-promotive bacteria. To provide direct evidence supporting the role of *Colpoda* spp. in disease control, the authors successfully isolated several *Colpoda* species from the rhizosphere soil. Among the different *Colpoda* species, *C. inflata*, which shows the highest sequence identity to *Colpoda* OTU67, showed a pronounced reduction in *R. solanacearum* cell numbers under sterile soil conditions, indicating that *C. inflata* directly consumes *R. solanacearum* cells, thereby conferring disease suppression. Further experiments in non-sterilized soil demonstrated that the application of 10^4^ cells/ml of *C. inflata* significantly reduced disease incidence while increasing the ratio of plant-beneficial bacteria, such as *Pseudomonas, Lysobacter*, and *Streptomyces*, thereby enhancing disease control effects. However, despite these promising results, its application into practical agricultural fields poses significant challenges. One critical question is how *C. inflata* selectively feeds on plant pathogenic (deleterious) bacteria while sparing beneficial ones. Another major pitfall is the large-scale cultivation of pure *Colpoda* spp. for field application. Furthermore, the underlying mechanisms driving the assembly of protective protists in the rhizosphere remain unclear. While it is known that the protist microbiome in the plant rhizosphere is intricately influenced by factors such as root exudates, rhizosphere microbiome composition, and soil conditions,^5^ the specific role of plant traits in attracting and assembling beneficial protists needs to be elucidated. One notable example of rhizosphere protist assembly influenced by plants can be seen in corn (*Zea mays*), where the root cap plays a crucial role, particularly at the root tip. When the root cap is removed, there is a significant decline in *Cercozoa* protists, which are important for regulating plant genes involved in immunity, such as jasmonate-induced protein and endoglucanase 1. The loss of *Cercozoa* weakens the plant defense system, destabilizes the rhizosphere microbiome, and eventually increases the plant’s susceptibility to pathogen invasion. Recent advances in metabolomics and transcriptomics could provide valuable insights into these processes, helping to clarify the complex interactions that contribute to effective disease suppression in agricultural systems.

In the context of biological weapons, the new concept of applying helper bacteria to inhibit pathogen-promotive bacteria while promoting pathogen-suppressive bacteria, along with the use of synthetic microbial communities (SynCom), will be another game changer in combating bacterial wilt.^6,7^ In addition, to avoid the pitfalls encountered with traditional single-microbe-based biological agents, it is crucial to understand how these microbial applications impact indigenous microflora. Remarkably, the SynCom protected tomato plants from bacterial wilt as effectively as the chemical positive control, 1 mM benzothiadiazole, known for strong disease suppression.^6^ Interestingly, rather than directly antagonizing *R. solanacearum*, the SynCom activated the plant’s systemic immunity. These findings highlight the pivotal role of plant innate immunity induced by the SynCom in defending against bacterial wilt and suggest that leveraging indirect pathogen control through biological weapons holds significant promise for field applications. Similar to protists, the rhizosphere bacterial community is largely influenced by host root exudates, which include organic acids, sugars, amino acids, flavonoids, phytochemicals, phytosiderophores, and more.^8^ However, the detailed molecular mechanisms behind the assembly of beneficial microbiome are not yet fully understood and should be a focus of future studies.

## Chemical weapons: community modulation rather than eliminating single species

Beyond conventional methods, two novel chemical approaches have recently been reported. Li and colleagues utilized a derivative of the ubiquitous bacterial second messenger, 2‘,3’-cyclic guanosine monophosphate (2‘,3’-cGMP), to control key biological functions in *R. solanacearum*. The authors discovered that 2’,3’-cGMP was instrumental in regulating numerous virulence-associated genes in *R. solanacearum*.^9^ In addition, they identified a key protein named RSp0334, which catalyzes the conversion of 2’,3’-cGMP into (2’,5’)(3’,5’)-c-di-GMP. The in-frame deletion of the *RSp0334* gene led to significant alterations in bacterial physiology, with the accumulation of 2’,3’-cGMP within *R. solanacearum*. The excess 2’,3’-cGMP binds to the transcriptional regulator RSp0980, preventing its interaction with target genes. Consequently, tomato plants infected with *R. solanacearum* mutants lacking either the *RSp0334* or *RSp0980* gene showed a 25% and 32.5% reduction in bacterial wilt, respectively, compared to those infected with the wild-type strain. The impaired phenotypes were restored by gene complementation, highlighting the pivotal roles of RSp0334 and RSp0980 in the pathogenicity of *R. solanacearum*. Considering that several phytopathogens and clinical pathogens produce 2’,3’-cGMP and possess proteins akin to RSp0334 and RSp0980, these chemical weapons could potentially be applied to manage a wide range of bacterial genera.^9^ However, this study did not explore the efficacy of soil treatment with 2’,3’-cGMP under field conditions, leaving this an open area for further investigation.

In a parallel development, Wen and coworkers identified seven key metabolites – gluconolactone, inositol, lactic acid, maltose, mannose, ribose, and xylose – prevalent in the rhizosphere of healthy tomato plants.^10^ These substances are readily consumed by soil commensal microorganisms but not by *R. solanacearum*. The application of a prebiotic blend containing these metabolites resulted in a 32% reduction in bacterial wilt occurrence in tomato plants. While the rhizosphere microbiome typically exhibits robust stability, the introduction of *R. solanacearum* disrupted this equilibrium, reducing the diversity of soil commensal bacterial genera. However, the prebiotic cocktail preserved these bacterial populations by promoting the growth of rhizosphere commensal microbes while simultaneously inhibiting *R. solanacearum*. Furthermore, the prebiotic treatment led to significant functional alterations in the rhizosphere microbial communities, particularly enhancing carbon metabolism and autotoxin breakdown. The authors also validated the effectiveness of the prebiotic blend in preventing bacterial wilt in other Solanaceae crops, including pepper and eggplant, highlighting its broad applicability in agricultural settings. As SynCom is being explored as a cutting-edge biological toolkit, their combination with prebiotics in field applications could substantially boost their effectiveness. Taken together, these findings indicate that plants modulate protective rhizosphere microbiota by secreting chemical cocktails probably through root exudates, thereby establishing a defensive barrier against *R. solanacearum* within the same ecological niche.

More recently, Jang and coworkers introduce a new biological weapon referred to as “plant-induced bacterial gene silencing (PIBGS)” to control *Ralstonia pseudosolanacearum*, a member of bacterial wilt family complex.^11^ Technically, this method targeted RNA silencing of *Ralstonia* specific virulence genes by the plant-generated bacterial small RNAs. PIBGS demonstrated a protective effect on disease severity in the greenhouse. Collectively, bacterial RNA silencing-based control methods offer a precise management tool for targeting specific bacterial pathogen species while preserving the indigenous microbiota. However we cannot exclude the potential for off-target effects on natural populations.. To avoid the problem, the target genes for PIBGS need to be carefully selected using sophisticated computational evaluations of soil and rhizosphere microbiota genomes.

## Conclusion and outlook

The Green Revolution of the mid-20^th^ century significantly boosted global crop yields through the development of high-yielding varieties and the widespread use of synthetic fertilizers and pesticides. However, as we face the challenges of the 21^st^ century, with a rapidly growing human population, there is an urgent need for a new Green Revolution. This time, however, we must learn from the unintended consequences of synthetic fertilizer and pesticide use and focus on developing sustainable strategies to increase crop yields. To achieve effective disease control with minimal adverse effects on human health and the environment, it is crucial to explore innovative disease management methods. In this *Commentary*, we highlighted new strategies for controlling bacterial wilt caused by *R. solanacearum*, a notorious soil-borne pathogen prevalent in tropical, subtropical, and monsoon areas (Figure 1). The novel approaches are designed not only to directly eradicate *R. solanacearum* but also to indirectly enhance the plant immune responses and modulate the rhizosphere microbiota. For example, the biological weapon involving predatory protists directly consumes *R. solanacearum* cells, while the use of the chemical weapon, 2‘,3’- cGMP, targets pathogenic bacteria without disrupting the indigenous microbiota. Such indirect disease control methods can offer a promising alternative to conventional chemical fertilizers and pesticides, minimizing their associated environmental and health side effects. We believe that the three recently discussed strategies for controlling bacterial wilt (physical, biological, and chemical) represent a significant advancement in the next-generation of crop diseases management. Farmers and scientists worldwide need to establish new integrated pest management (IPM) programs that combine these three innovative approaches with traditional methods, tailored to local environmental and economic conditions. In the face of global challenges, IPM will be key to sustaining agricultural productivity and ensuring food security through maintaining below disease potential level.

Nevertheless, the proposed integrated strategy requires further investigation into the intricate interactions between the host plant, the pathogen, and various control approaches (weapons). A comprehensive understanding of the interactions, particularly in relation to specific environmental and growth conditions, is essential for the effective implementation of these innovative methods. For instance, while epigenetically-encoded root immunity triggered by sound vibrations has shown potential, the possible trade-offs, such as energy costs to the plant under different stress conditions, have not been thoroughly examined. Additionally, the interplay between biological agents like predatory protists and chemical treatments such as 2‘,3’-cGMP may result in either synergistic or conflicting outcomes, depending on the dynamics of the soil microbiome. To address these complexities, future study should focus on developing systems biology approaches or computational models that simulate multi-way interactions between plants, pathogens, and control methods as well as environment such as climate changes. Such modeling could help minimize potential trade-offs and maximize synergistic effects in various environmental contexts. Moreover, long-term field studies will be necessary to evaluate the performance of these integrated approaches under real-world agricultural conditions.
